# Effectiveness of Socially Assistive Robots in Promoting Positive Emotional Responses and Alleviating Postoperative Pain Among Children: Quantitative Study

**DOI:** 10.2196/96800

**Published:** 2026-08-07

**Authors:** Fang-Yu Hsu, Yun-Hsuan Lee, Chih-Yuan Yang, Sue-hsien Chen, Shih-Ming Chu, Angela Shin-Yu Lien

**Affiliations:** 1 Graduate Institute of Nursing, College of Medicine, Chang Gung University, Taoyuan, Taiwan; 2 Department of Nursing Management, Chang Gung Medical Foundation Administration, Taoyuan, Taiwan; 3 Department of Artificial Intelligence, Chang Gung University, Taoyuan, Taiwan; 4 Artificial Intelligence Research Center, Chang Gung University, Taoyuan, Taiwan; 5 School of Nursing, Chang Gung University of Science and Technology, Taoyuan, Taiwan; 6 School of Nursing, College of Medicine, Chang Gung University, 259, Wenhua 1st Road, Guishan, Taoyuan, 33375, Taiwan, 886 2118800 ext 5186, 886 2118800; 7 Department of Pediatrics, Chang Gung Memorial Hospital, Taoyuan, Taiwan; 8 School of Medicine, College of Medicine, Chang Gung University, Taoyuan, Taiwan; 9 Department of Endocrinology and Metabolism, Linkou Chang Gung Memorial Hospital, Taoyuan, Taiwan

**Keywords:** socially assistive robot, postoperative care, facial expression analysis, pain, emotion, hospitalized children

## Abstract

**Background:**

Pain remains a critical issue among hospitalized children and may negatively affect postoperative recovery. In addition to pharmacological pain management, nonpharmacological approaches have been used to support pediatric care. Among these emerging approaches, socially assistive robots (SARs) may offer an opportunity to support children during hospitalization. However, limited evidence exists regarding the use of SARs in pediatric postoperative recovery and their influence on children’s emotional responses during child-robot interaction (CRI).

**Objective:**

This study aimed to examine changes in postoperative pain levels following a SAR intervention among hospitalized children. In addition, it aimed to explore emotional responses during CRI using automated facial expression analysis.

**Methods:**

A single-arm pre-post study was conducted in a pediatric surgical ward. Children recovering from surgery participated in a structured SAR intervention consisting of 3 phases: warm-up, educational video, and interactive engagement. Pain outcomes were assessed using the self-reported Wong-Baker FACES pain rating scale and the observer-rated FLACC (face, legs, activity, cry, and consolability) scale. Emotional responses were evaluated using automated facial expression analysis, which generated continuous emotional valence scores ranging from −1 (negative) to +1 (positive). Wilcoxon signed-rank tests were used to analyze pain outcomes, and Friedman tests were used to examine differences in emotional valence across intervention phases.

**Results:**

A total of 37 children were included in the pain outcome analysis, and 35 (95%) children were included in the emotional valence analysis after excluding participants with insufficient facial expression data. Significant reductions were observed in both self-reported and observed behavioral pain following the intervention. Self-reported pain scores decreased from a median of 6 (IQR 4-6) to 4 (IQR 2-4; *P*<.001), and FLACC scores decreased from a median of 3 (IQR 2-4) to 1 (IQR 1-2; *P*<.001). Emotional valence remained negative across all intervention phases. The Friedman test did not reach statistical significance across the 3 phases and showed a small effect size (*P*=.05).

**Conclusions:**

The SAR interventions may be associated with lower postoperative pain scores among hospitalized children. Although emotional valence did not significantly change during CRI, automated facial expression analysis was implemented and demonstrated the feasibility of continuous affective assessment in a real-world pediatric clinical setting. These findings support the potential use of the SAR interventions as a complementary strategy in pediatric postoperative care and provide preliminary evidence supporting the integration of real-time affective assessment into pediatric health care.

## Introduction

Hospitalized children frequently experience multiple stressors related to unfamiliar environments, invasive procedures, uncertainty, and pain [1-3]. Among these challenges, pain remains one of the most frequently reported and clinically significant concerns in the pediatric postoperative context [4,5]. Inadequately managed postoperative pain may adversely affect multiple aspects of recovery, including physical functioning, quality of life, sleep quality, oral intake, and cooperation with clinical care [4,6]. According to the International Association for the Study of Pain, pain is defined as “an unpleasant sensory and emotional experience associated with, or resembling that associated with, actual or potential tissue damage” [7]. This definition highlights that pain is a multidimensional phenomenon that involves both sensory and emotional components.

Pharmacological management is strongly recommended for pediatric postoperative pain alleviation [6,8]. Acetaminophen and ibuprofen are commonly recommended as first-line analgesics due to their established efficacy and safety when appropriately administered to children [6,9,10]. These oral analgesics commonly provide analgesic effects lasting approximately 4 to 6 hours and are frequently administered at scheduled intervals during postoperative recovery [9,10]. After tonsillectomy and/or adenoidectomy, additional analgesic medications, including opioid agents, may also be considered under careful clinical supervision [6,8]. Nevertheless, pediatric pain management is inherently multidimensional, incorporating both pharmacological and nonpharmacological strategies to optimize efficacy [4,11].

In clinical practice, distraction, play-based activities, child-friendly environments, education, social interaction, and family involvement are frequently integrated into pediatric care to alleviate pain, reduce negative emotional experiences, and facilitate coping during hospitalization [12-16]. Many nonpharmacological interventions have focused on distraction-based strategies that redirect attention away from painful or stressful stimuli, such as virtual reality, mobile apps, and games [11,17]. However, socially assistive robots (SARs) have emerged as a novel form of socially interactive supportive intervention [18-20]. SARs are robotic platforms specifically designed to assist users through social interaction rather than physical assistance [21]. Recently, SARs have been increasingly applied in health care settings to facilitate therapeutic engagement and emotional interaction [22,23].

Within the field of human-robot interaction (HRI), child-robot interaction (CRI) has attracted growing interest because children often respond to robots as engaging social agents rather than purely mechanical devices [18,24]. Accordingly, SARs have been used across pediatric settings, including outpatient clinics, hospital settings, and procedural environments, with a wide variety of robotic platforms and interaction designs [25-27]. Existing studies have primarily evaluated outcomes such as pain, anxiety, distress, and other negative emotional experiences, with generally favorable findings [28-31]. However, less attention has been paid to understanding how children’s emotional responses evolve throughout the CRI.

Understanding emotional responses during SAR interactions may provide additional insight into how such interventions influence children’s recovery experiences. Several validated self-report and observational instruments are currently available for assessing emotional and psychosocial outcomes in pediatric populations, including measures of fear [32], anxiety [33], distress [34,35], social behavior development [36], quality of life [37], and resilience [38]. These instruments provide important evaluations of children’s emotional and psychological outcomes across clinical settings [39,40]. However, children’s emotional responses may not be fully represented by a single discrete emotion during hospitalization in the real world [15,41]. Hospitalized children may simultaneously experience pain, uncertainty, and stress throughout the recovery process [1-3]. Therefore, emotions should be conceptualized as dynamic, continuously changing affective states rather than fixed categorical experiences. Within this framework, emotional valence reflects the overall affective direction of an individual’s emotional state, ranging from negative to positive, thereby providing a continuous measure of emotional experience over time [42,43]. The emotional valence dimension, which spans a positive-negative continuum, may provide a different perspective for exploring emotional responses during CRI [44].

Accordingly, this study aimed to examine changes in pain outcomes before and after a SAR intervention among hospitalized children recovering from surgery. In addition, the study explored emotional responses across CRI.

## Methods

### Study Design and Setting

This study used a single-arm pre-post experimental design to examine the effects of a SAR intervention on postoperative pain and emotional responses among hospitalized children. All interventions were conducted in the pediatric surgical ward of a tertiary medical center in northern Taiwan between 6 and 24 hours after surgery. A caregiver remained present throughout the intervention to support the child’s comfort and safety.

To minimize potential pharmacological confounding, the intervention was scheduled during a relatively stable period of postoperative analgesic coverage. Postoperative pain management followed the standard tonsillectomy care protocol at the hospital. All participants received acetaminophen as the primary postoperative analgesic 4 times a day. No participants received opioid medications or patient-controlled analgesia during the intervention period. Specifically, the intervention was delivered at least 3 hours after the most recent administration of oral acetaminophen and at least 1 hour before the next scheduled dose. This timing was selected because the analgesic effects of acetaminophen persist for 4 to 6 hours, allowing the intervention to occur during the mid-interval period rather than immediately after medication administration or immediately before the subsequent dose. In addition, no invasive medical procedures were performed within 30 minutes before the intervention.

### Participants

Eligible participants were consecutively recruited from the pediatric surgical ward during the study period based on the order of hospital admission. Children who met the inclusion and exclusion criteria were invited to participate in the study. The inclusion criteria were as follows: (1) aged 4 to 12 years, (2) hospitalized in the pediatric surgical ward, and (3) underwent tonsillectomy and/or adenoidectomy. Children were excluded if they demonstrated impaired consciousness, physical limitations that precluded interaction with the robot, or extensive facial wounds or dressings that could interfere with automated facial expression analysis.

The sample size estimation was conducted using G*Power (version 3.1.9.7) for a Wilcoxon signed-rank test. Assuming a medium effect size, a significance level of .05, and statistical power of 0.80, the estimated minimum sample size was 23 participants. To account for potential missing data and attrition, a total of 40 children were targeted for recruitment. The participant recruitment, retention, exclusions, and outcome analyses were reported.

### Intervention

**Figure 1.**
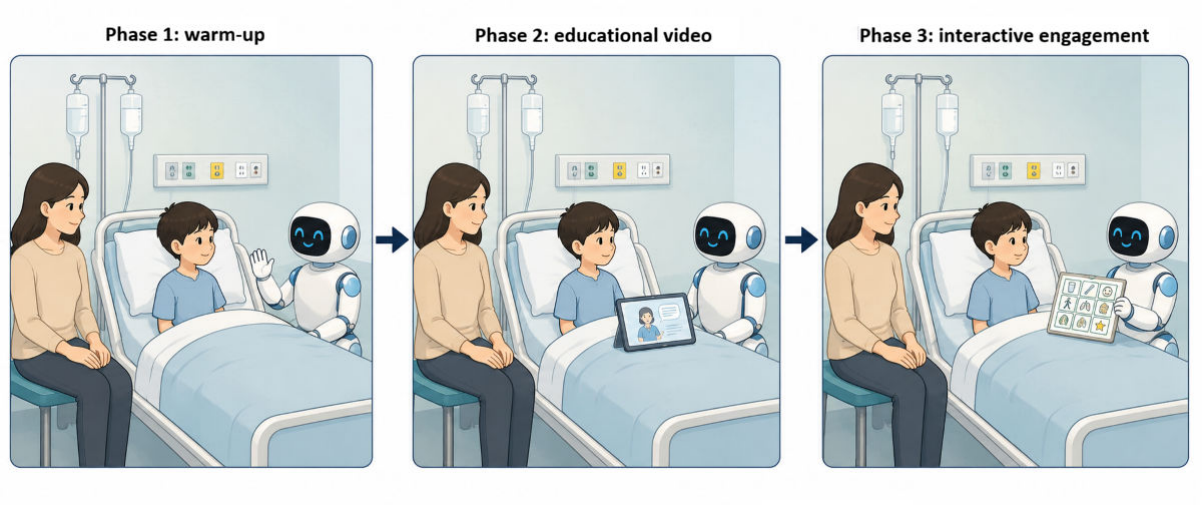
Overview of the 3-phase socially assistive robot intervention. Phase 1 (warm-up) focused on rapport building and baseline pain assessment. Phase 2 (educational video) delivered standardized postoperative self-care education using a child-friendly animated story. Phase 3 (interactive engagement) involved question-and-answer activities, reinforcement of educational content, supportive social interaction, and a Hero Sticker farewell activity.

The intervention consisted of 3 sequential phases: warm-up, educational video, and interactive engagement (Figure 1). The intervention was designed to establish rapport, deliver postoperative education, and reinforce engagement through structured CRI. To enhance intervention fidelity, a standardized database of dialogue scripts and robot facial expressions was developed before the intervention. Completion of all required intervention components was documented using a standardized checklist to ensure consistency across participants and study sessions (Table S1 in Multimedia Appendix 1).

Detailed descriptions of the content and representative interactions for each phase are presented in Table 1 and Table S2 in Multimedia Appendix 1. During the warm-up phase, the robot introduced itself and engaged the child in a brief, structured rapport-building conversation while guiding baseline pain assessment. The educational video phase consisted of a standardized postoperative education video presented on a tablet device. The video used a child-friendly narrative to introduce key postoperative self-care recommendations. During this phase, the robot remained physically present beside the child but did not engage verbally or nonverbally.

**Table 1.** Overview of the 3-phase socially assistive robots intervention.

Phases	Key activities	Interaction content
Phase 1: warm-up	Establish rapport and conduct baseline pain assessment	Robot self-introduction, rapport-building conversation, and baseline pain assessment
Phase 2: educational video	Deliver a standardized postoperative education video through a tablet	Story-based educational video covering hydration, medication adherence, cold therapy, dietary recommendations, and activity precautions
Phase 3: interactive engagement	Reinforce educational content, promote engagement, and facilitate closure	Educational question-and-answer activities, supportive feedback and encouragement, spontaneous child-initiated conversation, and a farewell ritual to acknowledge participation and provide relationship closure

In the interactive engagement phase, the robot reinforced educational content through question-and-answer activities, supportive feedback, and conversational exchanges. Children were also allowed to initiate spontaneous conversation, and responses were provided through a Wizard-of-Oz (WoZ) control interface while maintaining the overall intervention structure. The intervention concluded with a “Hero Sticker” farewell activity to acknowledge the child’s participation and provide a structured transition, facilitating closure of CRI and separation from the robot.

### WoZ Operation

**Figure 2.**
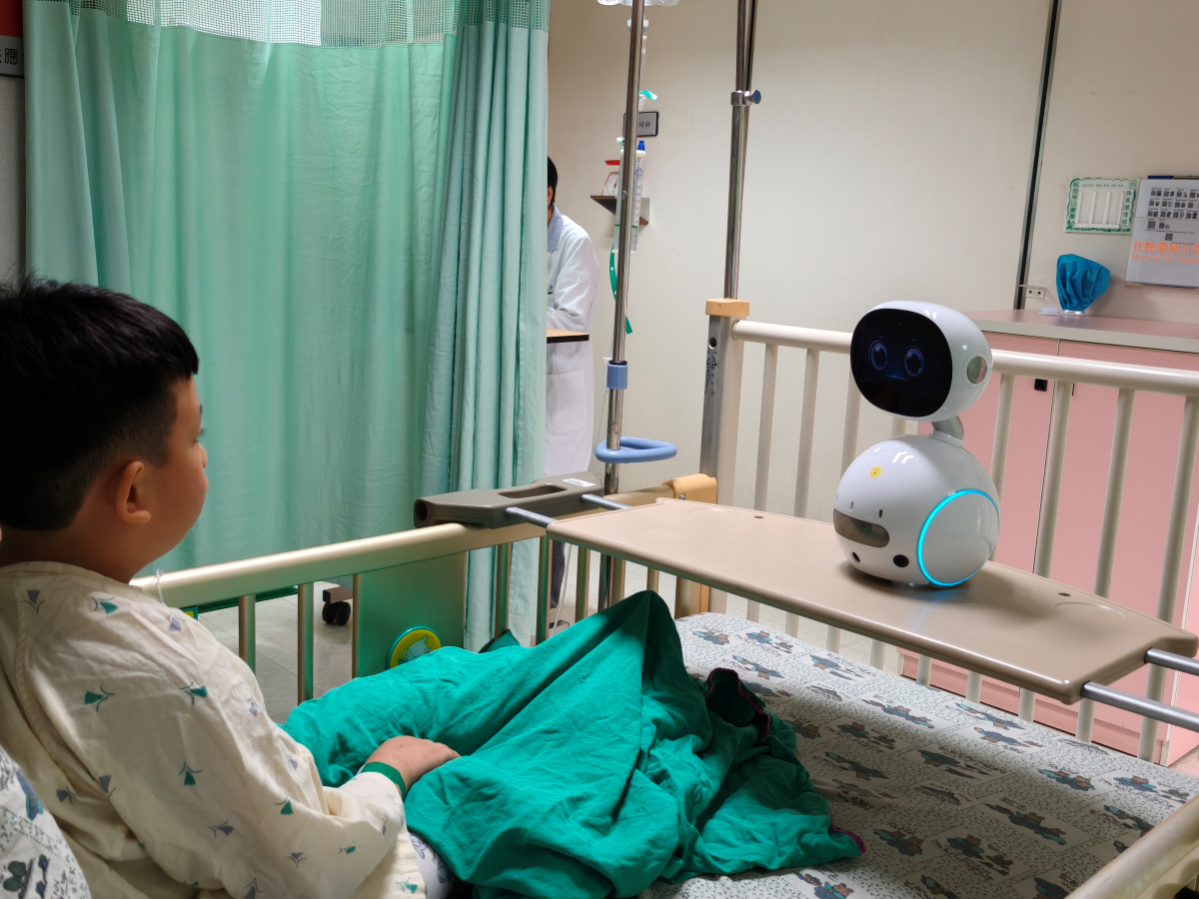
Intervention setup for the robot interaction. The child interacted with the robot at the bedside in the hospital room, while the robot was operated by a trained researcher using a Wizard-of-Oz control interface.

For safety and experimental control, the robot was operated under a WoZ operation [45]. As shown in Figure 2, the same trained operator, positioned outside the child’s visual field, remotely controlled the robot’s speech output and facial expressions via a computer interface (Figure S1 in Multimedia Appendix 1). The operator monitored a live audiovisual feed of the child to adjust responses while maintaining a standardized interaction structure. A second researcher remained at the bedside and did not verbally interact with the child unless necessary for safety. The operator received standardized training to ensure consistency in script implementation (Table S2 in Multimedia Appendix 1).

### Measures

#### Pain Assessment

Pain assessments were conducted at 2 time points: baseline (during the warm-up phase) and immediately after completion of the intervention. Both self-reported and observed behavioral pain were assessed. Self-reported pain was measured using the Wong-Baker FACES pain rating scale, which consists of 6 faces ranging from 0 (“no hurt”) to 10 (“hurts worst”) [46]. The robot provided standardized instructions, and participants selected the face or score that best represented their current pain level. Observed behavioral pain was measured using the FLACC (face, legs, activity, cry, and consolability) scale, a validated observational measure of pediatric pain [47,48]. Each of the 5 categories was scored from 0 to 2; total scores range from 0 to 10, with higher scores indicating greater pain severity. At each assessment time point, participants self-reported their pain using the Wong-Baker FACES pain rating scale, while a trained researcher simultaneously completed the FLACC assessment.

#### Emotional Valence

**Figure 3.**
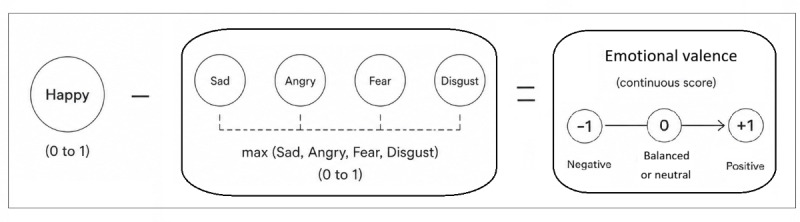
Calculation and interpretation of emotional valence. Emotional valence was computed as “Happy–max (Sad, Angry, Fear, Disgust)” using the standard FaceReader formula. Scores range from −1 to +1, with 0 representing relatively neutral emotional expression.

Emotional valence was selected as the primary affective indicator of emotional responses during the intervention. Emotional valence ranges from −1 to +1, where higher valence scores indicate more positive emotional expression and scores near 0 indicate relatively neutral emotional expression [49]. Following the standard computation, “Valence=Happy–max (Sad, Angry, Fear, Disgust),” emotional valence was calculated as the intensity of the Happy expression minus the highest intensity score among the negative emotions (sad, angry, fear, or disgust), as shown in Figure 3.

### Data Collection

All sessions were recorded using 1080p cameras positioned approximately 60° in front of the child to optimize facial visibility. Recorded videos were analyzed using FaceReader (version 10; Noldus Information Technology), an automated facial expression analysis system based on a deep artificial neural network that classifies facial expressions into basic emotional categories and computes emotional valence (Figures S2 and S3 in Multimedia Appendix 1). Because all participants were recruited in Taiwan, the East Asia model was used, as it was developed and validated on facial datasets from East Asian populations [50]. Previous studies have applied FaceReader across health care, psychological, and general settings and reported acceptable accuracy for automated facial expression assessment [51-54]. The software generated a continuous time-series dataset of emotional valence values from the recorded videos at a sampling rate of 10 frames per second. The exported dataset was subsequently segmented into the 3 intervention phases according to predefined time stamps: warm-up, educational video viewing, and interactive engagement.

### Quality Control

To ensure data quality, frames with significant facial occlusion (eg, turning away, touching the face, eating, or drinking), excessive head movement, or low facial detection confidence were automatically identified by FaceReader as invalid and excluded from subsequent analyses. Mean emotional valence scores for each intervention phase were calculated using only valid frames, with the denominator adjusted according to the number of successfully analyzed frames. To further ensure the reliability of facial expression measurements, the proportion of valid frames across the entire intervention session was calculated for each participant. Consistent with previous facial-analysis studies, a minimum detection threshold was applied [54]. Participants with fewer than a conservative 50% valid analyzable frames across all intervention phases were excluded from the final emotional valence analysis.

### Statistical Analysis

#### Overview

All statistical analyses were conducted using R (version 4.5.2; R Foundation for Statistical Computing). A 2-sided *P*<.05 was considered statistically significant. Descriptive statistics were used to summarize participant characteristics, intervention completion and duration, and the proportion of analyzable facial expression frames. Continuous variables are presented as means and SDs or medians and IQRs, as appropriate, whereas categorical variables are presented as frequencies and percentages.

#### Pain Outcomes Analysis

On the basis of the ordinal rating scales and within-subject assessments, the baseline and postintervention pain scores were compared using the Wilcoxon signed-rank test. For each comparison, the Hodges-Lehmann estimator of the median paired difference and corresponding 95% CIs were calculated. Effect sizes for Wilcoxon signed-rank tests were estimated using the rank-based effect size (*r*).

#### Emotional Valence Analysis

Participants with valid facial expression frames that met predefined quality control criteria were included in the emotional valence analyses. Mean emotional valence scores for each intervention phase were calculated for each participant. Differences in emotional valence across the warm-up, educational video, and interactive engagement phases were evaluated using the Friedman test for repeated nonparametric measurements. Effect sizes were estimated using Kendall W. When the Friedman test was statistically significant, post hoc pairwise comparisons were conducted using Wilcoxon signed-rank tests with Bonferroni-adjusted *P* values, when appropriate.

### Ethical Considerations

The study protocol was approved by the Chang Gung Medical Foundation Institutional Review Board (202500233B0C6001) on March 17, 2025. The recruitment period was from July 14, 2025, to September 15, 2025. Written informed consent was obtained from the parents or legal guardians of all participants before enrollment, and written assent was obtained from children aged ≥7 years. Participation was voluntary, and all collected data were stored in an anonymized form.

## Results

### Recruitment and Retention Flow

**Figure 4.**
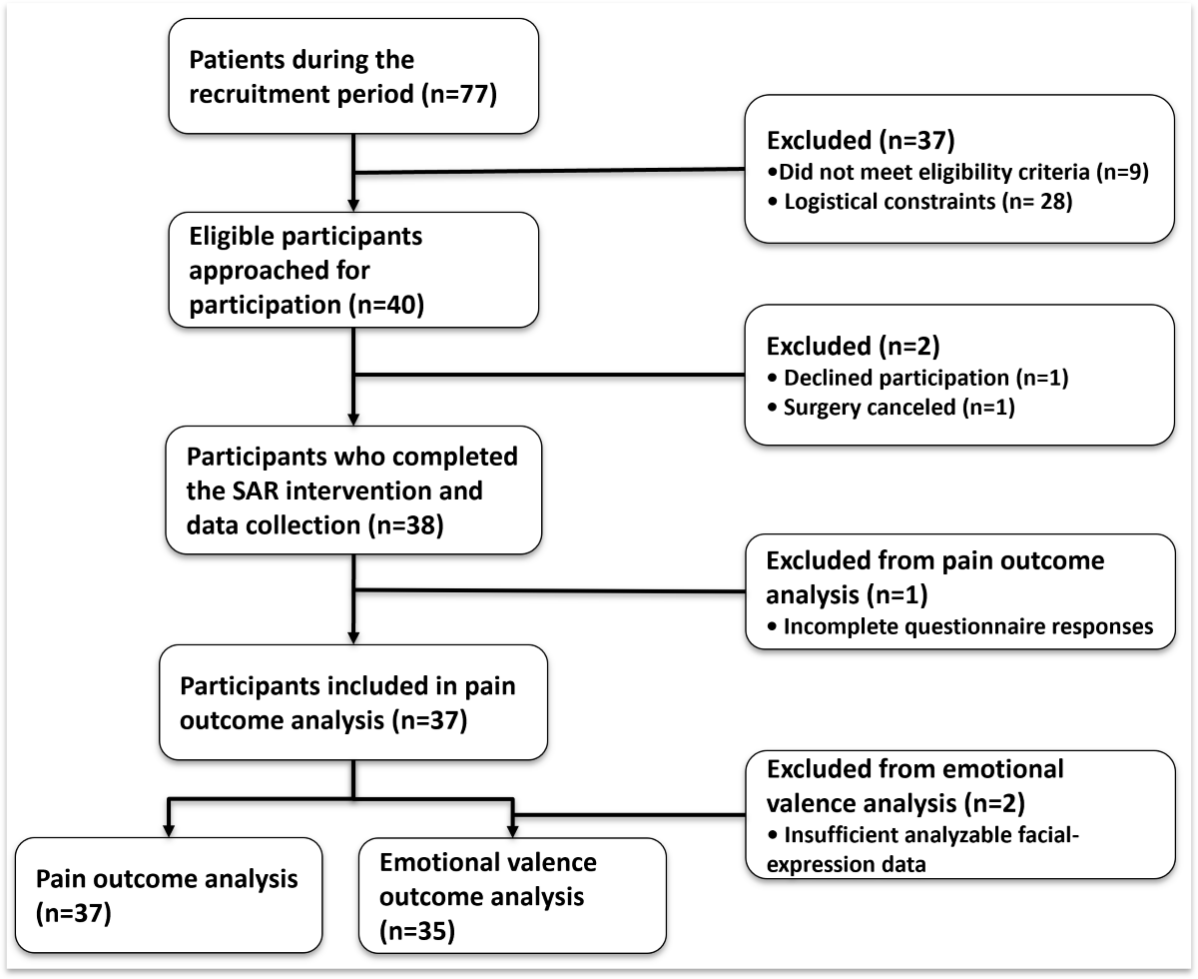
Participant recruitment, retention, and analysis flow. A total of 77 children were screened during the study period. Of the 40 eligible participants approached for participation, 38 completed the socially assistive robot (SAR) intervention and data collection. Final analyses included 37 participants for pain outcomes and 35 participants for emotional valence outcomes after predefined exclusions.

During the study period, 77 children undergoing tonsillectomy and/or adenoidectomy were screened for eligibility. Of these 40 children, 40 (100%) eligible participants were approached, and 38 (95%) completed the SAR intervention and data collection procedures. After excluding 1 (3%) participant with incomplete questionnaire data, 37 (97%) participants were included in the pain outcome analysis. For the emotional valence analysis, 2 (5%) participants were excluded because fewer than 50% of facial expression frames met predefined quality control criteria, resulting in a final sample of 35 (95%) participants. The participant recruitment and retention process is summarized in Figure 4.

### Participant Characteristics

Table 2 presents the characteristics of the 37 children included in the study. The mean age was 7.35 (SD 2.06) years, and most participants were boys (n=28, 75.7%) and were aged 7 to 12 (n=26, 70.3%) years. Previous hospitalization and surgical experience were reported by 45.9% (n=17) and 10.8% (n=4) of participants, respectively. More than half of the children (n=22, 59.5%) had prior exposure to robots, such as restaurant delivery robots, voice assistant devices, and household cleaning robots. The mean intervention duration was 17.33 (SD 7.46) minutes, and all participants completed the study protocol. Exploratory analyses indicated that baseline pain scores did not differ significantly according to age group, sex, or prior robot experience (Tables S3 and S4 in Multimedia Appendix 1).

**Table 2.** Demographic and clinical characteristics of the participants (N=37).

Characteristics	Participants
Age (years), mean (SD)	7.35 (2.06)
Age group (years), n (%)	
4-6^a^	11 (29.7)
7-12^b^	26 (70.3)
Sex, n (%)	
Boy	28 (75.7)
Girl	9 (24.3)
Experience of hospitalization, n (%)	
Yes	17 (45.9)
No	20 (54.1)
Experience of surgery, n (%)	
Yes	4 (10.8)
No	33 (89.2)
Experience with robots, n (%)	
Yes	22 (59.5)
No	15 (40.5)
Duration of intervention (minutes), mean (SD)	17.33 (7.46)
Completion of procedure, n (%)	37 (100.0)

a Mean age of participants in the 4- to 6-year age group is 5 (SD 0.77) years.

b Mean age of participants in the 7- to 12-year age group is 8.35 (SD 1.55) years.

### Pain Outcomes

**Figure 5.**
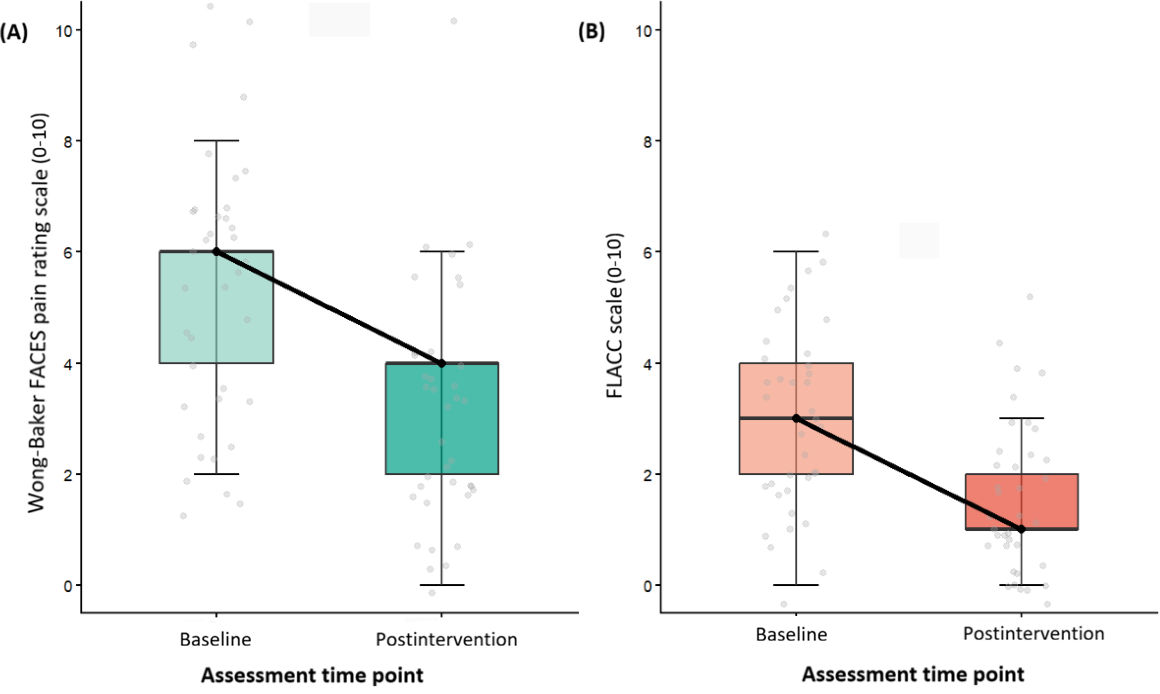
Changes in self-reported and observed behavioral pain following the socially assistive robot intervention (N=37). (A) Self-reported pain measured using the Wong-Baker FACES pain rating scale (0‐10) and (B) observed behavioral pain measured using the FLACC scale (0‐10). Boxplots represent the IQR. Gray dots represent individual participant scores at each assessment time point. Black points indicate median scores, and the connecting lines illustrate the overall change in median pain scores.

As shown in Table 3, both self-reported and observed behavioral pain decreased significantly following the SAR intervention. Wong-Baker FACES pain rating scale scores were significantly lower after the intervention than at baseline. The median difference was −3 (95% CI −4 to −2; *P*<.001), with a moderate effect size (*r*=0.48). Mean self-reported pain scores decreased from 5.24 (SD 2.37) to 3.19 (SD 2.23). Similarly, FLACC scores decreased significantly (median difference −2, 95% CI −2.5 to −1.5; *P*<.001), with a large effect size (*r*=0.54). Mean FLACC pain scores decreased from 3.03 (SD 1.66) to 1.57 (SD 1.35). Figure 5 illustrates the distribution of pain scores and individual participant changes across the assessment time points.

**Table 3.** Comparison of baseline and postintervention self-reported pain (Wong-Baker FACES pain rating scale) and observed behavioral pain (FLACC [face, legs, activity, cry, and consolability] scale).

Pain outcomes	Baseline, median (IQR)	Postintervention, median (IQR)	Median difference (95% CI)	*P* value	Effect size (*r*)
Wong-Baker FACES pain rating scale	6 (4-6)	4 (2-4)	−3 (−4 to −2)	<.001	0.48
FLACC scale	3 (2-4)	1 (1-2)	−2 (−2.5 to −1.5)	<.001	0.54

### Emotional Valence

A total of 2 (5%) participants were excluded because the proportion of analyzable facial expression frames was below the predefined 50% threshold, resulting in a final analysis of 35 (95%) participants. Among participants included in the emotional valence analysis, the mean proportion of analyzable video frames was 82.76% (SD 14.44%). Descriptive statistics for emotional valence scores across the 3 intervention phases are presented in Table 4. Mean emotional valence scores were negative across all 3 phases: −0.14 (SD 0.15) during the warm-up phase, −0.24 (SD 0.20) during the educational video phase, and −0.15 (SD 0.13) during the interactive engagement phase. The Friedman test did not reach statistical significance (*P*=.05) and showed a small effect size (Kendall W=0.084).

**Table 4.** Descriptive statistics of emotional valence across the 3 intervention phases (N=35)^a^.

Phases	Values, mean (SD)	Values, median (IQR)
Phase 1: warm-up	−0.14 (0.15)	−0.13 (−0.22 to −0.03)
Phase 2: educational video	−0.24 (0.20)	−0.20 (−0.33 to −0.09)
Phase 3: interactive engagement	−0.15 (0.13)	−0.10 (−0.23 to −0.07)

a Mean emotional valence scores were negative across all 3 phases.

**Figure 6.**
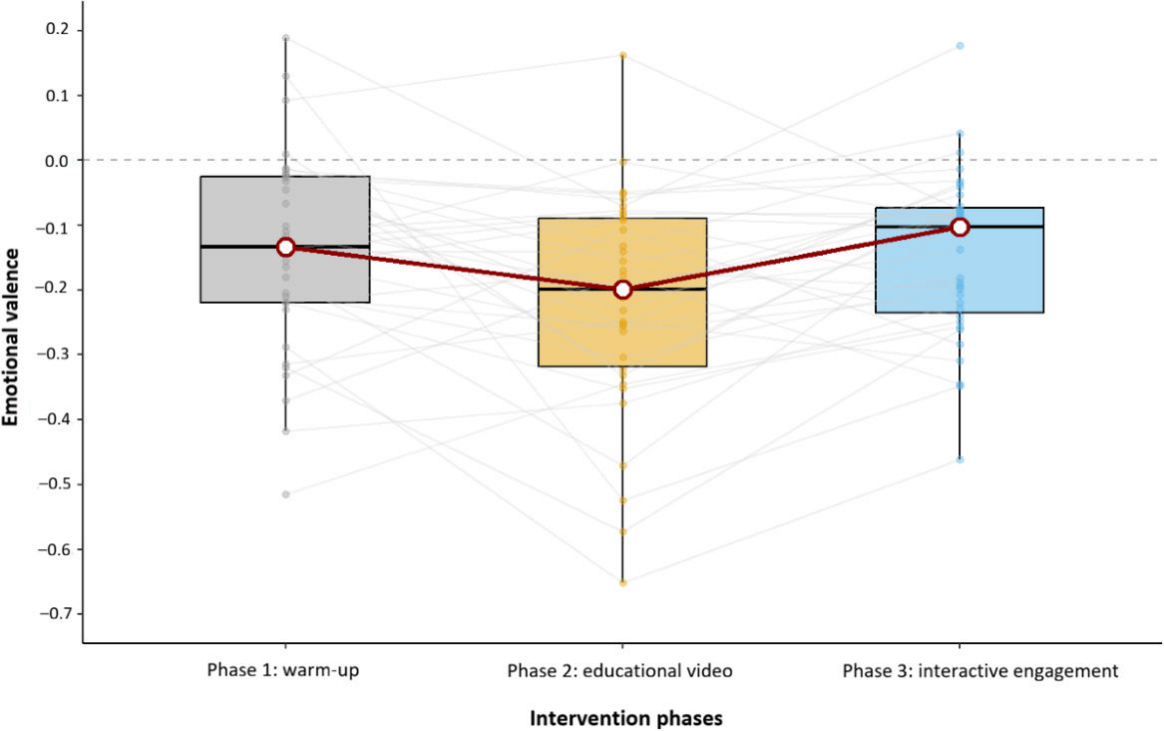
Distribution and individual trajectories of emotional valence across the 3 intervention phases (N=35). Boxplots represent the IQRs, with horizontal lines indicating median values and gray lines indicating individual participant trajectories across the intervention phases. The dashed horizontal line represents neutral emotional valence. Red circles indicate mean emotional valence scores for each phase, with the connecting line illustrating the overall trend in mean emotional valence during the intervention.

Although emotional valence demonstrated a V-shaped trajectory across the intervention phases (Figure 6), decreasing from the warm-up phase to the educational video phase, followed by an increase during the interactive engagement phase, the Friedman test did not reach statistical significance; therefore, the post hoc pairwise comparisons were not conducted.

## Discussion

### Principal Findings

This study examined pain outcomes and emotional responses during an SAR intervention among hospitalized children recovering from surgery. Significant reductions were observed in both self-reported and observed behavioral pain following the intervention. These findings suggest that participation in the SAR intervention was associated with lower pain scores during the postoperative recovery period.

In contrast, emotional valence did not significantly differ across the 3 intervention phases. Although emotional responses were continuously monitored throughout CRI using automated facial expression analysis, emotional valence remained predominantly negative across phases. Although pain scores decreased following the intervention, emotional valence did not significantly change across the intervention phases. These findings suggest that pain outcomes and emotional responses may represent distinct aspects of children’s postoperative recovery experiences.

### Pain Alleviation

Significant reductions were observed in both self-reported and observed behavioral pain following the SAR intervention. These findings are generally consistent with previous studies reporting favorable pain-related outcomes associated with SAR interventions among pediatric populations [26,55,56]. We acknowledge that spontaneous postoperative recovery, analgesic effects, and other unmeasured confounding factors may have contributed to the observed changes. However, the intervention and outcome assessments were completed within approximately 17 minutes and were scheduled to reduce the influence of immediate analgesic effects; the relative contribution of these factors cannot be distinguished in this study. Nevertheless, future controlled studies are needed to isolate the specific effects of the SAR intervention on postoperative pain outcomes.

Previous pediatric SAR studies have used a wide variety of robotic platforms and intervention approaches, including distraction, education, and cognitive behavioral strategies [56-59]. The variability in intervention designs and pain-related outcomes suggests that the components and design of the SAR interventions may warrant consideration.

The SAR intervention used in this study incorporated multiple components commonly applied in pediatric clinical care, including education, supportive communication, active engagement, and encouragement [16,60]. These approaches were delivered through a child-centered therapeutic process designed to facilitate participation throughout the intervention. Therefore, the effectiveness of the SAR interventions may depend not only on the robotic platform but also on the nature of the interaction delivered. This is consistent with the multidimensional nature of pain, which encompasses both sensory and emotional experiences [7].

From a clinical perspective, these findings suggest that pain-related outcomes may be influenced by multiple interaction components embedded within SAR interventions. Beyond providing distraction, SARs may offer a solution for delivering communication, education, engagement, and supportive interactions during pediatric postoperative care.

### Emotional Dynamics During CRI

Given the favorable findings of previous studies [28-30], an increase in emotional valence during the SAR interaction was expected. However, emotional valence remained within the negative range. It did not differ significantly across the 3 intervention phases, suggesting that the SAR intervention did not produce measurable changes in overall emotional valence during the observation period.

During hospitalization and the acute postoperative period, most children encountered multiple stressors, such as pain, physical discomfort, an unfamiliar environment, and uncertainty [1,5]. Therefore, a brief SAR interaction may not have been sufficient to produce measurable changes in overall emotional valence. Unlike previous studies that implemented SAR interventions during invasive procedures and reported reductions in negative emotional outcomes [26,29,61], participants in this study continued to experience ongoing pain. Furthermore, acute postoperative sore throat pain may have influenced facial movements and, consequently, the detection of emotional valence through facial expression analysis.

These findings highlight a potential distinction between pain outcomes and emotional responses. Although pain intensity may change over a relatively short time frame, emotional valence may be influenced by multiple contextual, physical, and individual factors [42,62]. Thus, the absence of significant changes in emotional valence does not necessarily indicate an absence of emotional benefit. Rather, it may reflect the complexity of emotional experiences during postoperative hospitalization and the limitations of relying solely on facial expression analysis. Nevertheless, the use of automated facial expression analysis in this study provided a feasible approach for examining emotional responses during HRI [44].

Taken together, these findings suggest that emotional responses during postoperative hospitalization may be more complex and less readily altered than pain outcomes during a brief SAR intervention. Although emotional valence did not significantly change across phases, continuous facial expression analysis may provide complementary insight into children’s affective experiences during CRI in clinical settings.

### Individual and Contextual Considerations

Children’s responses to the SARs varied considerably across individuals and appeared to be influenced by both personal and contextual factors [63]. Most participants demonstrated a willingness to interact with the robot, including touching, hugging, maintaining eye contact, and engaging in conversation. However, some caregivers reported that their children, who were typically cheerful and socially responsive, appeared more withdrawn during hospitalization because of postoperative discomfort. Despite these differences, all participants appeared to enjoy the “Hero Sticker” farewell activity (Figure S4 in Multimedia Appendix 1).

Informal observations during the intervention suggested that many children responded to the robot’s dynamic verbal and facial expressions. Children frequently mirrored expressive behaviors displayed by the robot, such as smiling or winking, and often appeared more engaged when verbal encouragement was accompanied by corresponding facial expressions (Figures S5 and S6 in Multimedia Appendix 1). These observations suggest that interaction quality may influence children’s participation during CRI.

Collectively, these observations highlight the complexity of CRI in clinical settings and suggest that children’s engagement with SAR interventions may be shaped by both individual and contextual factors. These considerations may be relevant when designing and implementing the SAR interventions in pediatric health care settings.

### Clinical Implications

The reduction in pain scores suggests that SAR interventions may serve as a complementary nonpharmacological strategy during pediatric postoperative recovery. The structured nature of the intervention may facilitate the delivery of postoperative education, supportive communication, and child-centered engagement. Although emotional valence did not significantly change, the successful implementation of automated facial expression analysis suggests that real-time affective monitoring may be feasible in real-world pediatric clinical environments.

Although this study was conducted in a pediatric surgical ward, the intervention approach may have broader applicability across pediatric health care settings. Because the intervention focused on communication, education, engagement, and supportive interaction, similar SAR approaches may be adapted to preoperative preparation, outpatient education, pediatric oncology units requiring repeated treatments and prolonged hospitalization, and other pediatric inpatient settings where emotional support and engagement are important. Collectively, these findings support the potential role of SARs as a complementary strategy for pediatric care while highlighting opportunities for future research across diverse clinical contexts.

### Limitations

Several limitations should be considered when interpreting these findings. First, the single-arm pre-post design without a control group limits the ability to determine the extent to which the observed changes were attributable to the SAR intervention rather than to other factors, such as spontaneous postoperative recovery, concurrent clinical care, previous experience with surgery and hospitalization, or other unmeasured confounding variables. In addition, the relatively small sample recruited from a single medical center may limit the generalizability of the findings. The predominance of boys in the sample reflected the epidemiological characteristics of pediatric tonsillectomy and adenoidectomy populations [64].

Second, automated facial expression analysis may not fully capture children’s emotional experiences during postoperative hospitalization. A total of 2 (5%) participants were excluded from the automated analysis because of insufficient analyzable facial expression data, and factors such as body movement, eating, drinking, and turning away from the camera may have affected the analysis.

### Future Directions

Future studies should evaluate SAR interventions using larger, more diverse samples, multicenter recruitment, and controlled study designs across a broader range of pediatric health care settings. Further research is also needed to develop and validate structured SAR intervention frameworks that integrate education, communication, engagement, and supportive interaction. Given the nonsignificant findings for emotional valence, future studies should incorporate multimodal emotional assessments, including self-report measures, behavioral observations, facial expression analysis, and physiological indicators, to better capture children’s emotional responses during SAR interventions.

Finally, this intervention was delivered using a WoZ approach, and further research should examine the feasibility and effectiveness of increasingly autonomous SAR systems. Determining whether autonomous robots can deliver standardized, responsive, and clinically meaningful interactions while maintaining safety, usability, and acceptance will be an important step toward the broader implementation of the SAR interventions in pediatric health care.

### Conclusions

This study examined pain outcomes and emotional responses during the SAR intervention among hospitalized children recovering from surgery. Following the intervention, both self-reported and observed behavioral pain scores decreased, while emotional valence remained relatively negative across the intervention phases. The findings suggest that the SAR intervention may serve as a complementary approach for pain experience during pediatric postoperative care. In addition, the implementation of automated facial expression analysis demonstrated the feasibility of continuously monitoring emotional responses in a real-world pediatric clinical environment. Collectively, these findings contribute preliminary evidence supporting the use of SAR interventions in pediatric health care and inform future research on SARs with multimodal emotional assessment.

## Supplementary material

10.2196/96800 Multimedia Appendix 1 Intervention checklist, Wizard-of-Oz operation procedures, interaction scripts, illustration of FaceReader analysis, additional statistical analyses, and photographs of the intervention implementation.

## Abbreviations

CRI: child-robot interaction
FLACC: face, legs, activity, cry, and consolability
HRI: human-robot interaction
SAR: socially assistive robot
WoZ: Wizard-of-Oz
