# Supplementary material for: Effectiveness of Socially Assistive Robots in Promoting Positive Emotional Responses and Alleviating Postoperative Pain Among Children: Quantitative Study
Source: J Med Internet Res. 2026 Aug 7;28:e96800. doi: 10.2196/96800 (PMC13450887; doi:10.2196/96800)
Supplement: Supplementary file 1 [file jmir-v28-e96800-s001.docx]

Supplementary

Table S1. Intervention Checklist

| No._______ | Sex____________  Age____________ | Recruitment date  ___________________ | Intervention date  ___________________ |
| --- | --- | --- | --- |
| Phase | Warm-up | Educational video | Interactive engagement |
| Time | ____: ____~  ____: ____ | ____: ____~  ____: ____ | ____: ____~  ____: ____ |
| Check | - robot self-introduction - rapport-building dialogue - pain assessment | - video viewed | - educational Q & A - sticker - pain assessment |
| Note | - Technical________ - Interruption______ - Other___________ | - Technical_________ - Interruption______ - Other____________ | - Technical_________ - Interruption______ - Other___________ |

Table S2. Representative Standardized Dialogue Script Used in the SAR Intervention. The dialogue shown below represents the standardized interaction framework. Additional spontaneous conversations initiated by participants were permitted and addressed through Wizard-of-Oz operation while maintaining the overall intervention structure.

| Phase or Purpose | dialogue |
| --- | --- |
| Warm-up | Hello! My name is Wizard XiaoBu.  I come from Planet Abu and I am here to help brave children defeat the throat pain monster. Are you ready?  Don't worry. The throat pain monster can be tough to beat, but I will teach you some magic tricks. Let's defeat it together, okay?  Do you know any ways to defeat the throat pain monster?"  Let's watch Susu's story. Just like you, Susu is learning how to defeat the throat pain monster. |
| Educational Content and Interactive Engagement | The first magic is Water Magic. Have you heard of it? Other children say it is super powerful!  Water Magic means drinking cool water or having cool things that flow like water.  The second magic is Ice Magic. Putting an ice pack on your neck may help your throat feel better.  The third magic is Magic Medicine.  Have you taken the special medicine that helps your throat feel better?  Magic Medicine works best when combined with Water Magic to defeat the throat pain monster.  Next, we have Magic Foods—foods that are cool, soft, and gentle on your throat.  Pudding is one kind of Magic Food. Can you think of any others?  Ice cream! Ice cream can help defeat the throat pain monster because it doesn't like cold things.  Grass jelly, jelly cups, and tofu pudding are also Magic Foods because they are cool and soft.  Remember, do not eat sour foods. Oranges, pineapples, lemons, and sour fruit juices should be avoided.  For the next few days, walk slowly, avoid running, and try not to speak too loudly.  What flavor of ice cream would you like?  Remember to swallow slowly and take small sips. Do not use a straw.  Keep practicing your magic skills to defeat the throat pain monster."  Keep practicing your magic skills! I will be cheering for you!  Amazing!  Wow! You learned that so quickly. Great job! |
| Educational Reinforcement and Question-and-Answer Activities | Let me see what magic you have practiced. Do you remember the different kinds of magic for defeating the throat pain monster?  Have you used Water Magic? Ice Magic? Magic Medicine? Have you eaten any Magic Foods?  Let me ask you a question. Which magic is most effective against the throat pain monster?  1. Cookies  2. Water Magic  3. Chicken nuggets  Another question. When using Water Magic, what should you do?  1. Drink through a straw  2. Take small sips  Very good! Here's a harder one. What can you eat right now?  1. Pudding  2. Orange juice  3. Magic Medicine  Excellent! Here's an even harder question. What should you do when you go home?  1. Play sports  2. Rest for one week  3. Shout loudly" |
| Farewell and Closure | It's almost time for me to return to Planet Abu. Whenever a child needs me, I will come back again!  I am very happy that we could play together today.  Now I am going to check how much your throat hurts.  Please choose your Bravery Sticker to show that you successfully defeated the throat pain monster.  See you next time. Goodbye! |
| Pain assessment | Here are six faces. The face on the left means no pain at all, and the face on the right means very, very severe pain. Which face looks most like how you feel right now? |

Figure S1. The control interface of the Wizard of Oz operation program.

Figure S2. Examples of output generated by FaceReader (version 10), including facial-expression classification, emotion probabilities, and emotional valence over time.

Figure S3. Examples of the generated valence-arousal graphic by FaceReader.

Table S3. Baseline WBFPRS demographic comparison

|  | group | p-value | significant |
| --- | --- | --- | --- |
| age | 4-6 vs 7-12 | 0.42 | NS^a^ |
| sex | Boy vs girl | 0.393 | NS |
| Robot experience | Yes vs no | 0.592 | NS |

^a^NS: No significant

Table S4. Baseline FLACC demographic comparison

|  | group | p-value | significant |
| --- | --- | --- | --- |
| age | 4-6 vs 7-12 | 0.473 | NS^a^ |
| sex | Boy vs girl | 0.438 | NS |
| Robot experience | Yes vs no | 0.683 | NS |

^a^NS: No significant

Figure S4. The real images of the “Hero sticker” activity at the end of intervention.

Figure S5. The mirroring behavior: wink.

Figure S6. The mirroring behavior: love heart.
